# Assessing Onchocerciasis Subcriticality from Pre-Intervention Cross-Sectional Surveys

**DOI:** 10.4269/ajtmh.19-0758

**Published:** 2020-05-26

**Authors:** John Daniel Kelly, Maria Rebollo Polo, Honorat Gustave Marie Zoure, Catherine E. Oldenburg, Jeremy D. Keenan, Travis C. Porco, Thomas M. Lietman

**Affiliations:** 1 Francis I. Proctor Foundation, UCSF, San Francisco, California;; 2 Department of Epidemiology and Biostatistics, UCSF, San Francisco, California;; 3 Institute for Global Health Sciences, UCSF, San Francisco, California;; 4 Expanded Special Project for Elimination of Neglected Tropical Diseases, World Health Organization, Brazzaville, Republic of Congo;; 5 Department of Ophthalmology, UCSF, San Francisco, California

## Abstract

Elimination of an infectious disease requires subcritical transmission, or a reproductive number less than one, and can be assessed with cross-sectional surveys conducted by neglected tropical disease programs. Here, we assess the distribution of onchocerciasis prevalence taken from surveys across sub-Saharan Africa before the initiation of ivermectin in mass drug administrations. Pre-intervention nodular palpation cross-sectional surveys were available from 15 countries in the Expanded Special Project for Elimination of Neglected Tropical Diseases (ESPEN) database. We determined whether the distribution of the prevalence over communities in an area was consistent with a geometric distribution, which previous studies have suggested indicates a subcritical disease. If not, we fitted a negative binominal distribution (hypothetically supercritical) or a mixture of two distributions: geometric (hypothetically subcritical) and Poisson (hypothetically supercritical). The overall distribution of community-level onchocerciasis prevalence estimates from the ESPEN dataset from 2005 to 2014 was not consistent with a geometric distribution. By contrast, data from several countries and parts of countries were consistent with the geometric distribution, for example, some areas within Nigeria and Angola. Even if the geometric distribution suggested pre-intervention subcriticality in more localized geographical areas, our model using pooled survey data of all geographic areas suggests that the entire pre-intervention prevalence does not fit a geometric distribution. Further work will be required to confirm the significance of a geometric distribution for onchocerciasis.

## INTRODUCTION

Neglected tropical disease programs typically use the mean incidence or prevalence over a geographical region to assess whether an intervention is required and whether control targets have been achieved. Thresholds allow programs to easily classify populations into those that have met the criterion. Those populations above the threshold may represent high, sustained transmission areas of endemic disease or the inevitable laggards in what will be a successful elimination program. A different criterion could be achievement of subcritical transmission, where the replacement number for infectious cases (*R*) is less than unity. If subcritical, infection would be expected to eventually be eliminated even without additional programmatic efforts. Identification of the prevalence threshold for subcriticality is a goal for programs, as this implies they are moving toward elimination. Although assessment could be performed with longitudinal surveys, subcriticality can also be assessed in cross-sectional surveys.^1–3^

Mathematical models have suggested that a geometric distribution might be expected when an infectious disease is subcritical.^4–6^ Cross-sectional surveys of diseases such as trachoma and leprosy are consistent with a geometric distribution across some areas that were expected to be headed toward elimination. Onchocerciasis is different from these two bacterial diseases for many reasons, including dependence on a geographically restricted vector. In response to these and other complexities, a range of mathematical models have been developed for onchocerciasis, including EPIONCHO and ONCHOSIM.^7,8^ The existing modeling literature does not address the question of inferring subcriticality from the distribution of cross-sectional data.

In May 2016, the WHO Regional Office for Africa launched the Expanded Special Project for Elimination of Neglected Tropical Diseases (ESPEN) to promote an integrated approach to disease elimination of onchocerciasis, lymphatic filariasis, trachoma, schistosomiasis, and soil-transmitted helminthiases.^9^ In 2018, as part of this program, the WHO created the ESPEN portal, an electronic platform designed to enable health ministries and stakeholders to share data related to disease surveillance (mapping community-level surveys) and geographic coverage of mass drug administration (MDA).^10^ The publicly available database contains thousands of onchocerciasis community surveys from a majority of the currently endemic sub-Saharan countries. Most of the surveys were conducted before the initiation of ivermectin in MDA.

Using the recently available ESPEN database, we assessed whether onchocerciasis among 15 African countries was subcritical before intervention. Specifically, we assessed whether cross-sectional surveys were consistent with a geometric distribution, which would be consistent with a reproduction number less than 1.^4,5^ If surveys were not consistent with a geometric distribution, we assessed whether the data could represent a mixture of subcritical and a portion of supercritical communities, reflecting hotspots in the region.

## METHODS

### Data.

The ESPEN database was accessed and included datasets from 15 African countries. These African countries represented those involved in the African Program for Onchocerciasis Control program. Periodic assessments of onchocerciasis programs were conducted as surveys according to the rapid epidemiological mapping of onchocerciasis (REMO) protocol. Communities were selected based on the location in relation to potential vector breeding sites, as determined on maps. Depending on the size of the selected community, the sample or subsample was considered to be those adults most at risk for onchocerciasis. If subsampling of larger communities was not feasible, then the community was replaced by a smaller one, where more representative sampling could occur. Cross-sectional surveys were conducted before intervention (MDA). During these rapid epidemiological assessment surveys, a sample of 30 to 50 adult men who were older than 20 years and had resided in the village for more than 10 years were examined for the presence of at least one fibrous nodule in the subcutaneous tissue, which was defined as a case of onchocerciasis. Then, the percentage of men with palpable nodules was calculated for each village, where a GPS coordinate was obtained from a central location in the village.^11^ Surveys were performed from 1989 to 2014, with those without an associated date known to have been performed well before 2001.

Note that some countries had multiple pre-intervention surveys conducted in more than one year; however, there were no longitudinal data collected during the pre-intervention period, meaning that surveys conducted in subsequent years were of previously unsampled communities.

We excluded surveys that were conducted after the intervention. We restricted our analysis to surveys that used nodular palpation as the diagnostic measure, instead of skin biopsy or Ov16 antigen testing, which were data collected during MDA. As the project used only de-identified publicly available data, the University of California, San Francisco, CHR-IRB considered this exempt from human subjects research protection.

### Parameterization of distributions.

The discrete distributions were parameterized such that the proportion infected would not, on average, be affected by the size of the survey. The scale parameter that was optimized was the mean proportional prevalence (*μ*). The two-parameter negative binomial was parameterized such that the shape parameter would equal one when the distribution represented the special case of a geometric distribution. With *Yj* denoting the number of infected individuals in the *j*th survey, we computed *P* (*Y*_*j*_ *= i*), the probability mass functions, as follows:

Geometric distribution with mean *n*_*j*_μ (and thus *P* = 1/(1+ *n*_*j*_*μ*)):

$$\frac{{\left(1-\frac{1}{1+n_{j}\mu }\right)}^{i}}{1+\mu n_{j}}\frac{{\left(1-\frac{1}{1+n_{j}p_{j}}\right)}^{i}}{1+n_{j}p_{j}}$$

Poisson distribution with mean *n*_*j*_*μ*:

$$\frac{e^{-np_{j}}{\left(n_{j}p_{j}\right)}^{i}}{i!}\frac{e^{-n_{j}\mu }{\left(\mu n_{j}\right)}^{i}}{i!}$$

Negative binomial distribution with mean *n*_*j*_*μ* and aggregation parameter *α*:

$$\;{\left(\frac{\alpha }{\alpha +\mu n_{j}\;}\right)}^{\alpha }{\left(1-\frac{\alpha }{\alpha +\mu n_{j}\;}\right)}^{i}\left(\begin{array}{c} \alpha +i-1\\ \alpha -1 \end{array}\right),$$

where *j* indicates the survey, μ is the proportion infected, and *n*_*j*_ is the number of individuals in the *j*th survey. Zero inflation was incorporated by having proportion *c* surveys necessarily zero. For the geometric–Poisson mixture, *c*_*1*_ was the proportion necessarily zero and *c*_*2*_ was the odds of being geometric as opposed to Poisson. Unless otherwise stated, calculations were performed in Mathematica 10.2 (Wolfram Research, Champaign, IL).

### Geometric.

To assess whether the distribution of the prevalence over communities in a country was consistent with a geometric distribution, we fitted a three-parameter, zero-inflated negative binomial model. More details about the use of geometric distributions and implications for subcriticality can be found elsewhere.^3,12^ Uncertainty was expressed using bootstrap percentile CIs. Because the geometric distribution is a negative binomial with a shape parameter of 1, we determined whether the 95% CIs for the negative binomial shape parameter included 1 by pairing the nearest neighbor villages (geographical distance) for bootstrap resampling. Based on the assumption that the distribution of infection approximates the quasi-stationary distribution in the subcritical circumstance,^6^ we approximate *R*_*0*_ by estimating μ/(1+μ), where μ is the parameter *P* of the fitted geometric distribution. We calculated the CI of *R*_0_ by bootstrapping the sample.

### Heterogeneity.

If the data were unlikely to have come from a zero-inflated geometric distribution, we fitted a 4-parameter mixture distribution, allowing for a proportion of subcritical communities (taken from a zero-inflated geometric distribution) and supercritical communities (taken from a Poisson distribution, representing random incidence of disease). The three models (zero-inflated geometric, zero-inflated negative binomial, and zero-inflated mixture of geometric and Poisson) were compared using the Bayesian information criterion (BIC). More details about the parameters for each distribution can be found in the Supplemental Materials.

### Subnational analysis.

To explore the distribution centered over a geographical region within a country and at each timepoint, we chose a grid of 27 evenly spaced internal points within the country’s minimum and maximum latitude and longitude. For each internal point, we randomly resampled surveys with inclusion probability based on the distance from the specific point (Gaussian kernel with a SD of 2° unless otherwise stated). All calculations were performed in Mathematica 11.1 (Wolfram Research).

### Time for prevalence to be reduced by 50%.

We estimate this time by the number of generations, *T*, of subcritical transmission needed for the disease to decline to half its current level, times the expected generation time. Here, *T* = ln (1/2)/ln *R*_*0*_. This assumes that there will be no intervention in these communities and an average duration of infection with the adult worm of 10 years.

## RESULTS

**Figure 1.**
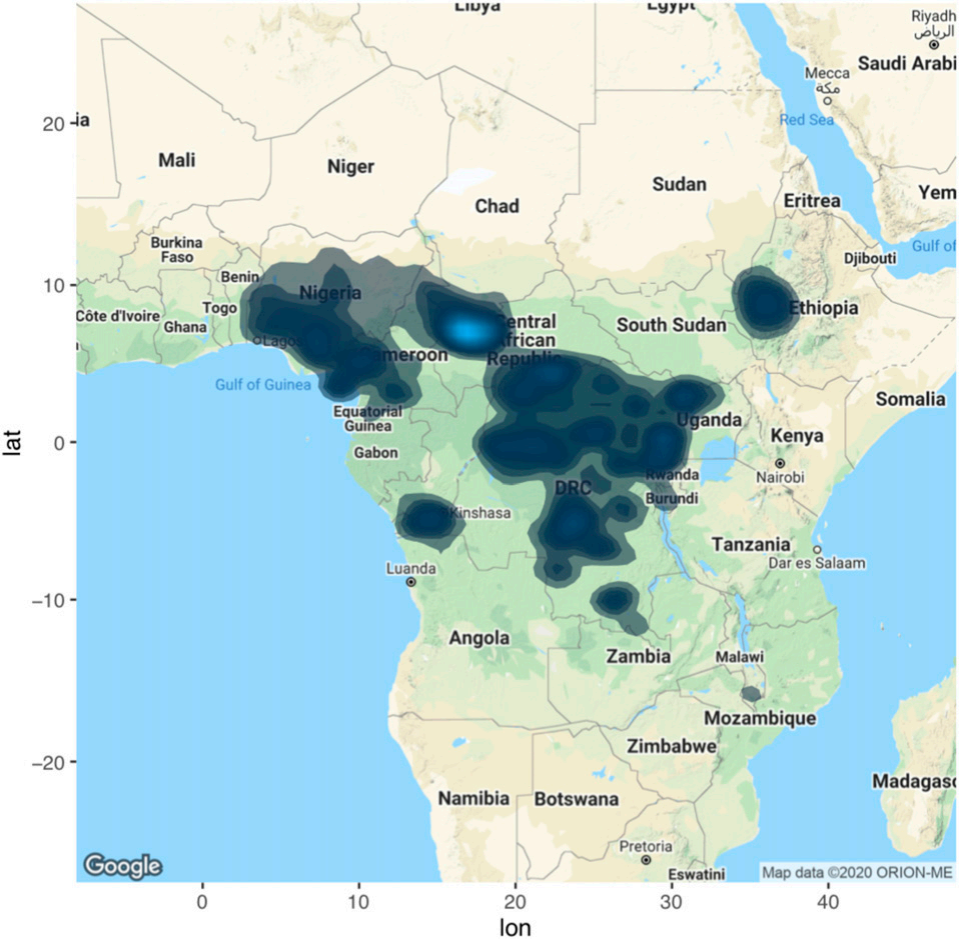
Map of the prevalence surveys. This figure appears in color at www.ajtmh.org.

The ESPEN database was accessed on May 10, 2018, and included 15,235 community-level prevalence survey results from 17 countries from 1989 to 2014. We excluded 1,876 surveys because they did not use nodular palpation as the diagnostic measure. The remaining 13,358 surveys were used in the overall analyses (Figure 1). We excluded an additional 4,154 surveys because there was no associated survey date available. The remaining 9,204 surveys were used in the country-level analyses.

**Figure 2.**
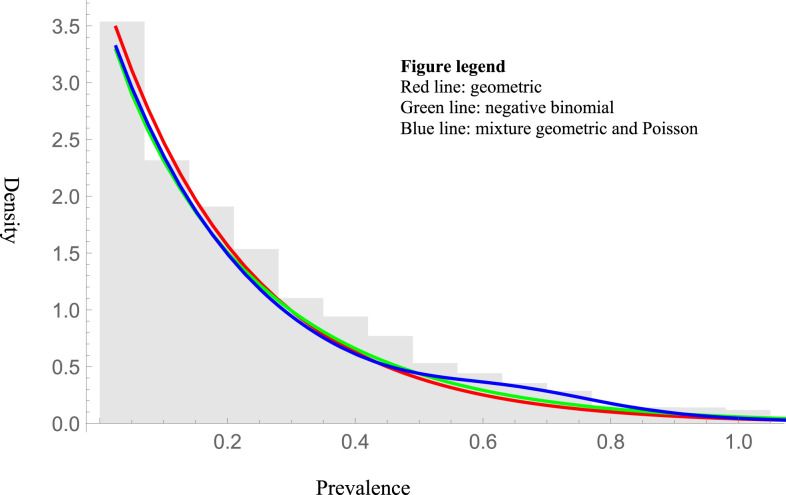
Histogram of the prevalence surveys from all available countries in sub-Saharan Africa over survey years, 1985–2014. The mixture of geometric and Poisson distributions had the lowest Bayesian information criterion. This figure appears in color at www.ajtmh.org.

As observed in Figure 2, available surveys were unlikely to have come from a geometric distribution (*P* < 0.001, zero-inflated geometric distribution goodness of fit testing). Although the shape parameter for the best-fit zero-inflated negative binomial distribution included 1 (Supplemental S1 Table), the mixture distribution had a far superior BIC (Table 1). This best-fit mixture distribution was a linear combination of 12% zero inflation (non-endemic), 83% geometric (hypothetically subcritical), and 5% Poisson distribution (hypothetically supercritical). As a result, 83% of communities are hypothetically subcritical. The average *R*_0_ of these communities was 0.91 (95% CI: 0.87, 0.95), which corresponds to a half-life of infection (time for prevalence to be reduced by 50%) of 70 years (95% CI: 68–72 years).

**Table 1:** Country-specific model comparisons

Country	Years	Surveys (#)	% Subcriticality for mixture distribution (95% CI)	Bayesian information criterion (distributions)	
All countries	not applicable	9,204	0.95 (0.94–0.97)	Geometric	56,496
				**Mixture**	**56,164**
				Negative binomial	56,551.8
Angola	2002 and 2011	762	0.89 (0.80–0.93)	Geometric	3,475.1
				**Mixture**	**3,470.1**
				Negative binomial	3,480.2
Burundi	2001 and 2013	186	0.96 (0.67–1.0)	Geometric	884.1
				Mixture	893.2
				**Negative binomial**	**880.5**
Cameroon	1993 and 2013	454	0.89 (0.78–0.96)	Geometric	2,718
				Mixture	2,723
				**Negative binomial**	**2,714.6**
Central African Republic	1999 and 2001	180	0.79 (0.72–0.88)	Geometric	891
				**Mixture**	**877.2**
				Negative binomial	896.2
Chad	2013	16	1.0 (1.0–1.0)	**Geometric**	**21.7**
				Mixture	27.3
				Negative binomial	23.9
Republic of the Congo	2003	93	0.84 (0.69–0.94)	Geometric	344.1
				**Mixture**	**335.8**
				Negative binomial	344.8
Democratic Republic of the Congo	2000 and 2014	3,727	0.85 (0.83–0.88)	Geometric	25,231.9
				Mixture	25,080.9
				**Negative binomial**	**25,070**
Cote d'Ivoire	2014	37	1.0 (1.0–1.0)	**Geometric**	**88.3**
				Mixture	95.5
				Negative binomial	91.2
Equatorial Guinea	1999 and 2013	247	0.71 (0.61–0.79)	Geometric	1,462.3
				**Mixture**	**1,402.1**
				Negative binomial	1,461.5
Ethiopia	2001 and 2012	644	0.70 (0.62–0.76)	Geometric	3,907.7
				**Mixture**	**3,829.7**
				Negative binomial	3,854.2
Gabon	1999 and 2014	78	0.23 (0-0.67)	Geometric	190.1
				Mixture	194.1
				**Negative binomial**	**189.6**
Malawi	1998	291	0.85 (0.74–0.89)	Geometric	755.6
				**Mixture**	**741.3**
				Negative binomial	745.3
Mozambique	2001 and 2007	291	0.72 (0.20–1.0)	**Geometric**	**429.3**
				Mixture	438.8
				Negative binomial	434.3
Nigeria	1989 and 2011	2,147	0.91 (0.88–0.97)	Geometric	12,511.6
				**Mixture**	**12,510**
				Negative binomial	12,516
Uganda	2008	51	0.84 (0.66–0.94)	Geometric	278
				Mixture	281.3
				**Negative binomial**	**276.7**

Bold font indicates the distribution with the lowest Bayesian information criterion.

### By nation and subnation.

**Figure 3.**
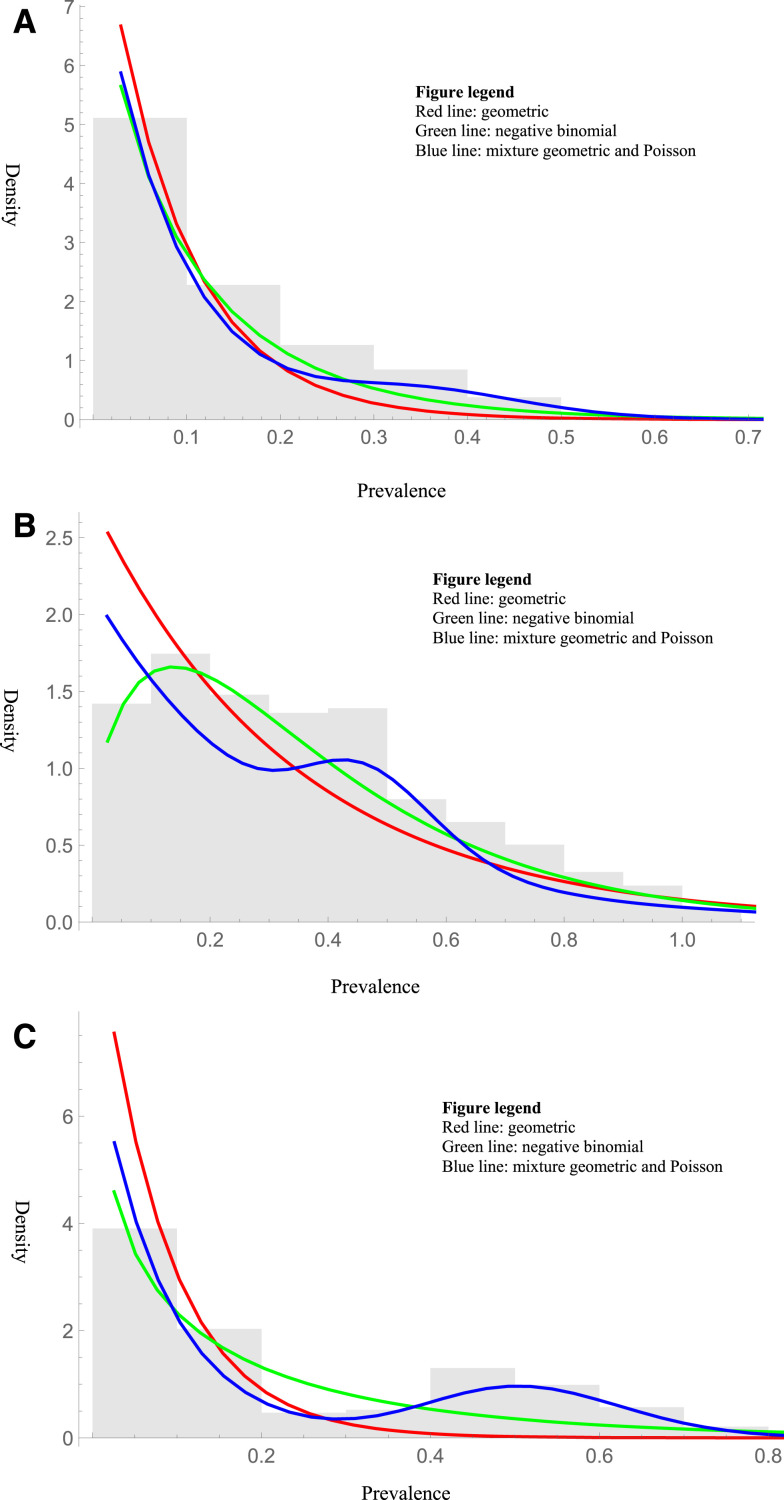
(**A**) Histogram of the prevalence surveys in Angola (2002–2011). The mixture of geometric and Poisson distributions had the lowest Bayesian information criterion (BIC). (**B**) Histogram of the prevalence surveys in Uganda (2008). The negative binomial distribution had the lowest BIC. (**C**) Histogram of the prevalence surveys in Equatorial Guinea (1999–2013). The mixture of geometric and Poisson distributions had the lowest BIC. This figure appears in color at www.ajtmh.org.

Countries had varying degrees of subcriticality (Table 1). Based on surveys conducted within a country, areas could be identified that were consistent with complete or near-complete subcriticality (Angola, Figure 3A). Some countries had data that were clearly not consistent with a geometric distribution (Uganda, Figure 3B). Others were consistent with a mixed geometric and Poisson (Equatorial Guinea, Figure 3C). Results describing the fit of each distribution can be found in the Supplementary Materials (Supplemental S1 and S2 tables).

**Figure 4.**
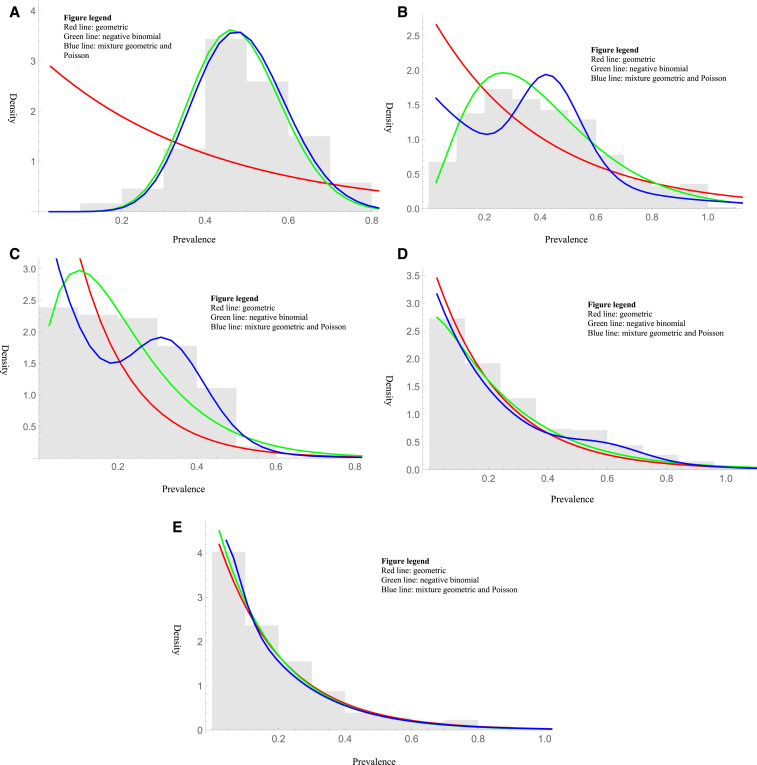
(**A**) Histogram of the prevalence surveys in Equatorial Guinea (2003). The negative binomial distribution had the lowest Bayesian information criterion (BIC). (**B**) Histogram of the prevalence surveys in the DRC (2009). The negative binomial distribution had the lowest BIC. (**C**) Histogram of the prevalence surveys in Ethiopia (2009). The negative binomial distribution had the lowest BIC. (**D**) Histogram of the prevalence surveys in Congo (Kinshasa) (2000). The geometric distribution had the lowest BIC. (**E**) Histogram of the prevalence surveys in Nigeria (1997). The geometric distribution had the lowest BIC. This figure appears in color at www.ajtmh.org.

It was not difficult to find areas within a country with varying degrees of subcriticality (Table 2, Figures 4A–E). Areas within Equatorial Guinea in 2003 (Figure 4A), the DRC in 2009 (Figure 4B), and Ethiopia in 2009 (Figure 4C) were not consistent with a geometric distribution, whereas areas within the DRC in 2000 (different areas from that in 2009; Figure 4D) and Nigeria in 1997 (Figure 4E) were consistent.

**Table 2:** Comparison of models for regions within specific countries

Country	Year	Surveys (#)	Central point (GPS: latitude, longitude)	% Subcritical for mixture distribution (95% CI)	Bayesian information criterion (distributions)	
Equatoria Guinea	2003	246	9.9, 3.1	0.02 (0.0, 0.06)	Geometric	2,128.5
					Mixture	1,425.5
					**Negative binomial**	**1,418.6**
Democratic Republic of the Congo	2009	3,726	26.4, 0.5	0.64 (0.61, 0.67)	Geometric	27,944.4
					Mixture	26,619.3
					**Negative binomial**	**25,161**
Ethiopia	2009	926	37.8, 6.0	0.64 (0.60, 0.69)	Geometric	5,448.2
					**Mixture**	**5,276.4**
					Negative binomial	5,294.5
Democratic Republic of the Congo	2000	4,398	17.0, −9.7	0.93 (0.92, 0.94)	Geometric	26,631.4
					**Mixture**	**26,339**
					Negative binomial	26,729.4
Nigeria	1997	2,146	11.6, 6.9	0.88 (0.83, 0.99)	**Geometric**	**12,613**
					Mixture	12,618.4
					Negative binomial	12,624.8

Bold font indicates the distribution with the lowest Bayesian information criterion (BIC). Note: There are two different areas where surveys were conducted in the Democratic Republic of the Congo (one in 2000 and the other in 2009).

## DISCUSSION

Onchocerciasis nodular palpation pre-intervention surveys from the overall ESPEN database were unlikely to have come from a geometric distribution, which mathematical models have suggested might be expected when an infectious disease is subcritical.^4–6^ Given that mass distribution of ivermectin had yet to begin in these places, we expected pre-intervention surveys to be inconsistent with the geometric distribution. However, we found some pre-intervention surveys were consistent with the geometric distribution. These areas are potentially subcritical. If so, elimination would eventually be achieved without further intervention.

A geometric distribution of community-level prevalence can be obtained by assuming subcriticality and infectiousness in a simple susceptible-infectious-susceptible transmission model. Particular assumptions about transmission may not be necessary. Any stochastic process where disease is being eliminated and future cases are proportional to current cases may result in a geometric distribution.^3,13^ Also, previous studies have shown that this distribution may be seen over larger geographical areas and with some heterogeneity. For trachoma and leprosy, a remarkable consistency with a geometric distribution has been observed, over a time period when the diseases were in the process of being eliminated.^1–3^ As a vector-borne disease, onchocerciasis exhibits more complex transmission dynamics than trachoma or leprosy, and published onchocerciasis models have accounted for the vector, age, treatment, vulnerabilities, infection intensity, and other factors.^7,14–17^ These transmission dynamic differences may be one reason that many of our findings were inconsistent with a geometric distribution.

Another explanation may have to do with survey sampling techniques. Rapid epidemiological mapping of onchocerciasis samples high-risk villages first and then secondary villages nearby. Inside each selected village, REMO assesses a targeted sample of 30–50 men older than 20 years who engage in rural work and have been living in the community for at least 10 years. Distributions based on this biased population of communities and individuals might not be expected to conform to a geometric, even if a population-based survey would. Trachoma and leprosy indicators are population based. In simple models, the prevalence of communities where infection persists approaches a geometric distribution as infection disappears^4,5,18,19^; however, this is a quasi-stationary distribution—an equilibrium contingent on a community having at least one infectious case. Trachoma and leprosy dynamics may be slow enough that distributions are close to this equilibrium.^1–3^ That may not be true of other diseases such as onchocerciasis.

The modeling approach in this analysis was simple. However, it should be noted that the hypothesis that the prevalence of a disappearing infectious disease would approach a particular distribution should hold more than a wide variety of assumptions. Making inferences from a distribution may be more difficult in practice than simply assessing whether a community has reached a prevalence threshold. Here, we have assumed that the distribution found in surveys near in time and place to our point of interest can be used to approximate the probability space (ensemble average) at that point—a form of spatiotemporal ergodicity. But too much heterogeneity of communities over space and time may make this assumption unrealistic.

We have allowed for the possibility of heterogeneity between communities in a number of ways. The geometric distribution reveals the amount of heterogeneity expected from a stochastic subcritical infectious process, even if all communities had identical transmission. The relatively heavy tail of the geometric (compared with that of, e.g., the Poisson or binomial distributions) implies that a proportion of higher prevalence communities would be expected even in a successful treatment program. Heterogeneity between communities was also included in the negative binomial model. A shape parameter of less than one allows more dispersion than seen in the geometric distribution, and a shape parameter more than one allows less. A beta-geometric distribution would allow for a varying rate of elimination in different areas, although that was not performed in this analysis as the limited database might not have supported the necessary additional parameter. Note that a beta-geometric would also be monotonically decreasing but allow for a tail heavier than the geometric. Fitting a mixture of a geometric and a Poisson distribution modeled heterogeneity by allowing for two distinct subsets of communities, providing an estimation of the proportion subcritical and critical, respectively. Clearly, distributions more complicated than the one-parameter Poisson used here could be considered for modeling supercritical areas. Although our approach is simple, a large class of models would be expected to approach this behavior as the infection is disappearing.

The approach taken in this report suffers from a number of additional limitations. With onchocerciasis, subcriticality may not be the only explanation for observing a geometric distribution of prevalence across communities. Unlike trachoma and leprosy, vector heterogeneity plays an important role in onchocerciasis. Communities closer to blackfly breeding sites are far more vulnerable. If the probability of infection were to decrease with the distance along a river from breeding sites or decrease away from infested rivers, then a J-shaped distribution similar to the geometric could be possible, even with stable disease. Further geographic and longitudinal studies could assess which of these two competing mechanisms is producing distributions close to the geometric.

Assessing distributions requires a great deal of data. However, the data were sufficient to exclude the geometric over the entire area. At the country level (Figure 3), we included all available data even when the associated dates were not available, yet the number of surveys provided less power to distinguish between different distributions. Maximum likelihood estimations for the dispersion parameter of the negative binomial distribution can be biased with smaller sample sizes. Here, most of the estimations had 100s or even 1000s of samples, minimizing any such bias. For examples of distributions seen over more focused areas within a country (Figure 4), we did not correct for the multiple comparisons—note that to do so would be anti-conservative, as it would expand the CIs and make it easier to not reject the geometric. Thus, these examples should just be taken as case studies. Even with enough data, the approach allows categorization of areas but not individual communities. The use of palpable nodules was considered a sufficient proxy for the prevalence of onchocerciasis infection and was the primary way of determining the prevalence during the pre-intervention period of REMO. Nodules, however, are poorly sensitive and specific for onchocerciasis,^20^ which are further dependent on regional and other variations.^21^ Not enough publicly available data currently exist for testing diagnostic techniques, such as skin biopsy or Ov16 antigen, to do this analysis, although hopefully these can be analyzed in the future.

Typically, control has been defined by a prevalence or incidence threshold, such as less than 5% active trachoma in children at the district level, or less than 1:10,000 newly prevalent cases for leprosy. However, in onchocerciasis, targeted sampling strategies such as REMO are used to assess control. These criteria are relatively easy to implement and can be applied from individual surveys. However, assessing for control would in theory require examining every available cluster. The alternative definition of subcriticality offers complementary information. A reproduction number greater than unity (criticality) would imply a lack of control with current interventions, or a potential hotspot. The knowledge that an area has become subcritical can help inform programs in other ways. For example, when a district has reached a threshold, we expect that approximately 37% (1/*e*) will remain greater than that threshold. But these may not represent increased transmission potential, just stragglers in a controlled area.

Subcriticality not only implies a measure of control but also suggests that a program is on the path toward elimination. If seen over a region, it could suggest that elimination is a reasonable program goal. If seen over the most affected continent, for example, trachoma in Africa or leprosy in South Asia, it could suggest the path toward eradication. Subcriticality does not however imply that elimination or eradication would occur quickly. Without effective agents against macrofilaria, even subcritical onchocerciasis areas may take decades to control. Whereas here we used surveys of nodular palpation, in the future, surveys using more specific tools such as OV16 antigen or PCR tests could easily be used. Although subcriticality should be a goal of control programs, the expected timescale of decline must also be considered.

## materials and tables

Supplemental materials
