# Supplementary material for: Assessing Onchocerciasis Subcriticality from Pre-Intervention Cross-Sectional Surveys
Source: Am J Trop Med Hyg. 2020 May 26;103(1):287–94. doi: 10.4269/ajtmh.19-0758 (PMC7356432; doi:10.4269/ajtmh.19-0758)
Supplement: Supplementary file 1 [file tpmd190758.SD1.pdf]

Supplemental Materials

Each survey has an associated numerator and denominator. All distributions were parametrized by the proportion of the population infected, facilitating combining surveys with different denominators. For the geometric distribution, parameters include the proportion of zero-inflation, c, and the mean/total of the geometric distribution, mu. Parameters for the zero-inflated negative binomial included the proportion of zero-inflation, c, the shape parameter n, and the mean/total, mu. Parameters for the zero-inflated mixture of geometric and Poisson included the proportion of zero-inflation, c1, the odds of the mixture distribution being geometric, c2, the mean of the geometric distribution, mu1, and the mean/total of the Poisson distribution, mu2. 95% CIs in Table S1 were estimated using bias corrected bootstrap of nearest neighbor pairs (to correct for spatial clustering).

S1 Table. Findings by country over survey years.

**Country**	**Geometric distribution (c: zero-inflation; µ: mean/ total) (95% CI)**	**Mixture of geometric and Poisson distribution (µ1: mean/ total of geometric; µ2: mean/ total of Poisson; c1: zero-inflation; c2: odds of geometric /mixture) (95% CI)**	**Negative binomial distribution parameters (µ: mean/ total; c: zero-inflation; n: shape) (95% CI)**
All countries	µ: 0.23 (0.22-0.23)<br>c: 0.12 (0.11-0.14)	µ1: 0.20 (0.19-0.21)<br>µ2: 0.67 (0.492-0.75)<br>c: 0.12 (0.10-0.13)<br>c2: 19.0 (14.1-27.0)	µ: 0.23 (0.22-0.23)<br>c: 0.11 (0.09-0.13)<br>n: 0.95 (0.90-1.02)
Angola	µ: 0.10 (0.095-0.12)<br>c: 0.093 (0.046-0.14)	µ1: 0.071 (0.054-0.12)<br>µ2: 0.31 (0.04-0.34)<br>c: 0.05 (0-0.10)<br>c2: 7.46 (3.71-12.07)	µ: 0.10 (0.09-0.11)<br>c: 0.039 (0.0-0.13)<br>n: 0.82 (0.65-1.19)
Burundi	µ: 0.090 (0.063-0.13)<br>c: 0.16 (0.085-0.26)	µ1: 0.075 (0.023-0.12)<br>µ2: 0.34 (0.028-0.80)<br>c: 0.15 (0.03-0.27)<br>c2: 15.45 (0.0->100,000)	µ: 0.08 (0.05-0.10)<br>c: 0 (0.0-0.12)<br>n: 0.53 (0.43-0.84)
Cameroon	µ: 0.18 (0.16-0.20)<br>c: 0.040 (0.0096-0.083)	µ1: 0.16 (0.13-0.19)<br>µ2: 0.33 (0.027-0.64)<br>c: 0.04 (0.01-0.09)<br>c2: 9.58 (5.44-25.14)	µ: 0.17 (0.15-0.19)<br>c: 0 (0.0-0.022)<br>n: 0.75 (0.65-0.88)
Central African Republic	µ: 0.22 (0.18-0.27)<br>c: 0.41 (0.30-0.51)	µ1: 0.10 (0.059-0.14)<br>µ2: 0.47 (0.44- 0.52)<br>c: 0.39 (0.25-0.51)<br>c2: 2.23 (1.40-4.62)	µ: 0.22 (0.16-0.29)<br>c: 0.41 (0.21-0.55)<br>n: 0.99 (0.55-1.80)
Chad	µ: 0.0034 (0.0022-0.46)<br>c: 1.33*10^-7 (0-1.0)	µ1: 0.0034 (0.0022-0.46)<br>µ2: 0.45 (0.45-0.45)<br>c: 1.33 (0.0-1.0)<br>c2: >100,000 (0->100,000)	µ: 0.003 (0.002-0.90)<br>c: 0 (0.0-1.0)<br>n: >100,000 (>100,000->100,000)
Congo (Brazzaville)	µ: 0.099 (0.064-0.14)<br>c: 0.39 (0.24-0.55)	µ1: 0.022 (0.0080-0.051)<br>µ2: 0.28 (0.23-0.33)<br>c: 0.12 (0.02-0.36)<br>c2: 4.38 (1.19-13.01)	µ: 0.06 (0.04-0.20)<br>c: 0 (0.0-0.73)<br>n: 0.31 (0.21-1.41)
Congo (Kinshasa)	µ: 0.32 (0.31-0.33)<br>c: 0.049 (0.038-0.059)	µ1: 0.28 (0.27-0.30)<br>µ2: 0.53 (0.48-0.65)<br>c: 0.05 (0.04-0.06)<br>c2: 5.60 (4.66-7.30)	µ: 0.34 (0.33-0.35)<br>c: 0.093 (0.082-0.10)<br>n: 1.62 (1.52-1.74)
Cote d'Ivoire	µ: 0.0081 (0.0025-0.015)<br>c: 0.35 (0.049-0.76)	µ1: 0.0081 (0.0025-0.015)<br>µ2: 0.45 (0.45-0.45)<br>c: 0.35 (0.05-0.76)<br>c2: >100,000 (>100,000->100,00)	µ: 0.005 (0.001-0.016)<br>c: 0 (0.0-0.74)<br>n: 0.38 (0.0->100,000)
Equatorial	µ: 0.20 (0.18-0.25)	µ1: 0.070 (0.053-0.085)	µ: 0.19 (0.16-0.25)

Guinea	c: 0.12 (0.081-0.19)	µ2: 0.49 (0.47-0.52)<br>c: 0.04 (0.0-0.09)<br>c: 2.62 (1.65-3.62)	c: 0.021 (0.0-0.12)<br>n: 0.63 (0.46-0.95)
Ethiopia	µ: 0.21 (0.19-0.22)<br>c: 0.056 (0.034-0.10)	µ1: 0.16 (0.14-0.18)<br>µ2: 0.32 (0.29-0.35)<br>c: 0.07 (0.04-0.12)<br>c2: 2.00 (1.42-2.74)	µ: 0.22 (0.21-0.24)<br>c: 0.12 (0.010-0.16)<br>n: 2.17 (1.79-2.69)
Gabon	µ: 0.022 (0.018-0.028)<br>c: 6.62*10^-9 (0.0-4.67*10^-8)	µ1: 0.0093 (0.0-0.027)<br>µ2: 0.028 (0.021-0.35)<br>c: 0.06 (0.0-0.31)<br>c2: 0.42 (0.42-2.42)	µ: 0.03 (0.02-0.08)<br>c: 0.16 (0.0-0.34)<br>n: 11.44 (0.0->100,000)
Malawi	µ: 0.14 (0.12-0.15)<br>c: 0.71 (0.61- 0.76)	µ1: 0.071 (0.046-0.11)<br>µ2: 0.21 (0.18-0.24)<br>c: 0.72 (0.61-0.76)<br>c2: 1.00 (0.63-1.71)	µ: 0.16 (0.14-0.17)<br>c: 0.75 (0.65-0.79)<br>n: 3.69 (2.51-6.69)
Mozambique	µ: 0.014 (0.0088-0.021)<br>c: 0.34 (0.019-0.51)	µ1: 0.011 (0.000043-0.043)<br>µ2: 0.024 (0.0019-0.14)<br>c: 0.22 (0.0-0.54)<br>c2: 97.45 (96.73->100,000)	µ: 0.01 (0.01-0.02<br>c: 0 (0.0-0.63)<br>n: 0.45 (0.17->100,000)
Nigeria	µ: 0.15 (0.14-0.16)<br>c: 0.0053 (0.0023-0.026)	µ1: 0.14 (0.13-0.15)<br>µ2: 0.19 (0.17-0.20)<br>c: 0.02 (0.001-0.035)<br>c2: 11.72 (8.65-32.72)	µ: 0.15 (0.14-0.16)<br>c: 0.027 (0.0017-0.051)<br>n: 1.12 (1.00-1.28)
Uganda	µ: 0.24 (0.15-0.33)<br>c: 0.38 (0.24- 0.56)	µ1: 0.090 (0.0-0.24)<br>µ2: 0.64 (0.33-0.81)<br>c: 0.34 (0.03-0.52)<br>c2: 2.97 (0.96-8.78)	µ: 0.16 (0.07-0.33)<br>c: 0.068 (0.0-0.40)<br>n: 0.28 (0.067-1.27)

S2 Table. Findings within country by survey year.

Country	Year / Surveys	Geometric distribution (c: zero-inflation; μ: mean/ total) (95% CI)	Mixture of geometric and Poisson distribution (μ1: mean/ total of geometric; μ2: mean/ total of Poisson; c1: zero-inflation; c2: odds of geometric /mixture) (95% CI)	Negative binomial distribution parameters (μ: mean/ total; c: zero-inflation; n: shape) (95% CI)
Equatorial Guinea	2003 / 246	μ: 0.48 (0.47-0.50)<br>c: 0.0 (0.0-0.0)	μ1: 0.27 (0.25-0.43)<br>μ2: 0.48 (0.47-0.50)<br>c: 0.0 (0.0-0.0)<br>c2: 0.02 (0.01-0.07)	μ: 0.48 (0.46-0.49)<br>c: 0.0 (0.0-0.0)<br>n: 113.48 (0.0- >100,000)
Congo (Kinshasa)	2009 / 3726	μ: 0.38 (0.38-0.38)<br>c: 0.0 (0.0-0.0)	μ1: 0.38 (0.37-0.39)<br>μ2: 0.37 (0.36-0.39)<br>c: 0.0 (0.0-0.0)<br>c2: 1.87 (1.62-2.09)	μ: 0.38 (0.38-0.39)<br>c: 0.01 (0.01-0.02)<br>n: 3.14 (3.01-3.32)
Ethiopia	2009 / 926	μ: 0.19 (0.18-0.20)<br>c: 0.0 (0.0-0.0)	μ1: 0.0093 (0.0-0.027)<br>μ2: 0.028 (0.021-0.35)<br>c: 0.06 (0.0-0.31)<br>c2: 0.42 (0.42-2.42)	μ: 0.21 (0.20-0.23)<br>c: 0.10 (0.07-0.12)<br>n: 2.83 (2.51-3.27)
Congo (Kinshasa)	2000 / 4398	μ: 0.25 (0.24-0.26)<br>c: 0.20 (0.18-0.21)	μ1: 0.20 (0.19-0.21)<br>μ2: 0.75 (0.71-0.79)<br>c: 0.19 (0.17-0.20)<br>c2: 10.91 (9.03-13.77)	μ: 0.24 (0.23-0.25)<br>c: 0.16 (0.15-0.18)<br>n: 0.80 (0.74-0.86)
Nigeria	1997 / 2146	μ: 0.15 (0.14-0.16)<br>c: 0.07 (0.06-0.08)	μ1: 0.14 (0.13-0.15)<br>μ2: 0.19 (0.17-0.20)<br>c: 0.02 (0.001-0.035)<br>c2: 11.72 (8.65-32.72)	μ: 0.15 (0.14-0.16)<br>c: 0.02 (0.02-0.05)<br>n: 0.82 (0.78-0.90)

Data source: Available at http://espen.afro.who.int/
